# Gender difference in eye dominance in patients with schizophrenia and normal controls

**DOI:** 10.1192/j.eurpsy.2025.623

**Published:** 2025-08-26

**Authors:** K. V. Akabalieva

**Affiliations:** Medical University, Sofia, Bulgaria

## Abstract

**Introduction:**

Hemispheric imbalance might underlie the genesis of psychosis. In patients with schizophrenia left eye dominance was higher than in control subjects. The aim of the study is to investigate sexual differences in left eye dominance as a biological marker of neuronal dysontogenesis in schizophrenic patients and control subjects. This study is part of a larger investigation project on the intriguing relations between six groups of markers of neuronal dysontogenesis - left-handedness, left-footedness, left-eyedness, minor physical anomalies, digit ratio, and cognitive (attention and memory) deficit.

**Objectives:**

Altered cerebral lateralization is a key trait found in many neurological and psychiatric disorders. This study investigates the gender difference in eye dominance between patients with schizophrenia and controls.

**Methods:**

The study was conducted in the Clinic of Psychiatry at the University Hospital in Sofia and the State Psychiatric Hospital in Radnevo. The sample included 98 (56 men, 42 women) consecutively admitted schizophrenic inpatients with a mean age 34.45 years for men and 42.20 years for women and 82 control subjects (30 men, 52 women) with a mean age 34.70 for men and 44.50 years for women. Three tests for eye dominance were administered as performance tasks- Looking through a monocle, Hole test and Porta test.

The non-parametric Mann-Whitney test was used to analyse the data.

**Results:**

The mean left-eyedness is significantly higher in schizophrenic patients than in control subjects in the three eye tests: Looking through a hole - .81 vs. .39, p<.001, over two times increase; Looking through a monocle - .78 vs. .39, p<.002, two times increase; Porta test - .86 vs. .41, p<.002, over two times increase.

In male subjects the difference between schizophrenia and controls reaches statistical significance for Looking through monocle (p=.037), but does not reach statistical significance for Hole test (p=.196) and Porta test (p=.077). In contrast, all female intra-gender comparisons between schizophrenia and controls reach statistical significance- Looking through monocle (p=.013) and Porta test (p=.015) reach statistical significance at p<.05, while Hole test at p=.001.

**Conclusions:**

The patients with schizophrenia have significantly higher mean left-eyedness than the controls in both sexes, but this difference is much more pronounced in women than in men for all three eye tests. Altered eye dominance in patients with schizophrenia is related to a prenatal maldevelopment and exhibits clear gender difference.

**Disclosure of Interest:**

None Declared

